# Thymic Stromal Lymphopoietin (TSLP) promotes human eosinophil-basophil in situ hemopoieisis, and its secretion from human nasal epithelium is a function of TSLP genotype

**DOI:** 10.1186/2045-7022-5-S4-O3

**Published:** 2015-06-26

**Authors:** Judah Denburg, Claudia Hui, Helen Neighbour, Delia Heroux, Loubna Akhabir, Andrew Sandford

**Affiliations:** 1 McMaster University, Department of Medicine, Hamilton, Ontario, Canada; 2 St. Paul’s Hospital, University of British Columbia, Vancouver, British Columbia, Canada

## Background

Allergic diseases are characterized by tissue eosinophilic and basophilic inflammation. There is substantial evidence that this particular inflammatory profile results from the migration to tissues of a common eosinophil-basophil (Eo/B) progenitor that undergoes a differentiative process regulated by local cytokines, termed in situ hemopoiesis. We therefore investigated the role and mechanisms of TSLP involvement in human Eo/B in situ hemopoiesis in relation to atopy. Also, since candidate gene and genome-wide association studies have identified ‘protective’ associations between the single nucleotide polymorphism (SNP) rs1837253 in the TSLP gene and risk for allergy, asthma and airway hyper-responsiveness, we evaluated the secretion of TSLP protein from primary nasal epithelial cells (NEC) in relation to rs1837253 genotype.

## Methods

Peripheral blood CD34+ cells derived from atopic and nonatopic individuals were stimulated with and/or IL-3, IL-5, or GM-CSF and assessed for Eo/B colony forming units (CFU) in both methylcellulose and nasal epithelial/CD34+ cell air-liquid interface (ALI) co-cultures, and cytokine surface receptor expression by flow cytometry. Genotyping was performed using a commercially available TaqMan® genotyping assay.

## Results

∘ TSLP preferentially enhanced IL-3/TNFα-dependent Eo/B CFU from CD34+ cells;

∘ Eo/B CFU and TSLPR expression were significantly increased in atopic-derived CD34+ cells post TSLP- (p<0.05) and IL-3/TSLP-stimulation (p<0.001), compared to non-atopic-derived CD34+ cells;

∘ In progenitor/NEC ALI co-cultures, secreted TSLP was biologically active, and sufficient to induce the differentiation of CD34+ cells into Eo/B CFU;

∘ There was decreased TSLP secretion by nasal epithelial cells obtained from heterozygous (CT; 1.8-fold) and homozygous rs1837253 TSLP minor allele (TT; 2.5-fold) individuals, compared to homozygous ‘wild-type’ individuals (CC; p<0.05).

## Conclusions

These studies further support the concept of in situ hemopoiesis and point out a previously unrecognized, critical role for TSLP in the development of allergic upper airway inflammation and its role in Eo/B differentiation, a link between adaptive and innate immunity. Novel functional genomics of TSLP show that the rs1837253 polymorphism may be directly involved in the regulation of TSLP secretion, providing explanations for both genetic association studies and recently reported positive clinical trials targeting TSLP in allergic asthma.

